# An Accurate Projector Calibration Method Based on Polynomial Distortion Representation

**DOI:** 10.3390/s151026567

**Published:** 2015-10-20

**Authors:** Miao Liu, Changku Sun, Shujun Huang, Zonghua Zhang

**Affiliations:** 1 State Key Laboratory of Precision Measuring Technology and Instruments, Tianjin University, Tianjin 300072, China; E-Mails: excalibur_s@tju.edu.cn (M.L.); sunck@tju.edu.cn (C.S.); 2 School of Mechanical Engineering, Hebei University of Technology, Tianjin 300130, China; E-Mail: huangsj@hebut.edu.cn

**Keywords:** aberration compensation, projector calibration, structure light measurement, 3D printing, photodiode, subpixel measurement

## Abstract

In structure light measurement systems or 3D printing systems, the errors caused by optical distortion of a digital projector always affect the precision performance and cannot be ignored. Existing methods to calibrate the projection distortion rely on calibration plate and photogrammetry, so the calibration performance is largely affected by the quality of the plate and the imaging system. This paper proposes a new projector calibration approach that makes use of photodiodes to directly detect the light emitted from a digital projector. By analyzing the output sequence of the photoelectric module, the pixel coordinates can be accurately obtained by the curve fitting method. A polynomial distortion representation is employed to reduce the residuals of the traditional distortion representation model. Experimental results and performance evaluation show that the proposed calibration method is able to avoid most of the disadvantages in traditional methods and achieves a higher accuracy. This proposed method is also practically applicable to evaluate the geometric optical performance of other optical projection system.

## 1. Introduction

Digital projectors are widely used in several fields such as projecting displays, structure light measurement [1] and 3D printing systems [2]. Many methods are created to improve the performance of the digital projector like acquiring higher accuracy, higher speed and more convenience. Due to the different specified applications and the non-ideal optical systems, digital projectors should be calibrated before working. Up to now, many image lens calibration approaches are proposed to meet the requirements of various applications. Compared with the projecting display application, some applications (e.g., 3D printing, structure light measuring and projecting instructing) require higher accuracies of the projecting image. Precise and reliable methods should be applied on these accuracy sensitive applications.

The optical structure of a projection system can be considered as a reverse structure of an imaging system, where the object plane and image plane are exchanged and the entire optical path is reversed. Thus, the existing calibration methods for cameras are not directly applicable to calibrate the projector. Some projector calibration methods have been proposed to decrease the effect of camera distortion. A pre-calibrated CCD camera was used to capture the projected pattern on a white plate in order to estimate the distortion parameters of the projection lens [3]. However, the projector’s final calibration result is severely affected by the pre-calibration accuracy of camera in this approach. In another approach, the mark points on the calibration plate are used as the position reference in the projector’s image space so it does not rely on camera pre-calibration. Several images are captured by moving the camera or the projector, and then the homography created by the mark points between projector and camera was applied to calculate the parameters of the projector [4,5,6]. In some other approaches, the projection patterns and the marks on the calibration plate are recorded and combined in a single image. The homography can be obtained through pattern analysis without moving the projector and camera [7]. In recent study, a beam splitter is applied to build up a virtual coaxial structure. By using a calibration plate with small ring markers on the surface and combined with the phase-calculation algorithm, the corresponding distortion of each projector pixel is able to be calculated independently. [8,9] However, conventional distortion representation is still applied. Some methods which operate on the entire camera-projector systems apply a joint calibration procedure. They are more convenient in some specific applications (e.g., multi-projector combination system and some kinds of projector-camera 3D imaging systems) [10,11,12,13]. In summary, all the existing methods are based on three steps: First, display the projected images on a diffusion plate; second, use the camera to capture the pattern image which is projected on the diffusion plate; third, analyze the captured images with the conventional imaging lens distortion model. All of these procedures obtain the projection lens’ distortion parameters or complete a joint calibration for the whole camera-projector system. However, all of these methods have the following disadvantages that result in accuracy decreases:

1. Photogrammetry will introduce extra errors into the final measurement result of the projection lens.
2. The accuracy of the sub-pixel algorithm is limited by the noise, resolution and dynamic range of the camera.
3. Due to the defect of the diffusion plate, the reflectogram is not constant at different orientations and positions.
4. Although with the similar structure, the conventional distortion parameters for the imaging lens are not suitable for the projection lens, because high optical efficiency and horizontal posture of the projector is the main concern in designing the projection lens. Large residues will be introduced into the final measurement result by using conventional distortion model.

To alleviate such disadvantages, a new projector calibration approach is proposed in this paper. The principle of this approach is to find the relationship between the ideal point on the image plane and the corresponding point on the object plane. Photodiodes (PDs) are applied to detect the rays emitted from a projector. The position of an ideal point on the image plane is defined by the center of the PD when it stops at a known position. The subpixel coordinates on an object plane, which correspond to the PD’s position on the image plane, can be obtained by scanning procedure. The entire field of the projection lens’ distortion property can be acquired by repeating the scanning procedure.

Compared with the method based on image processing, neither camera nor diffusion plate is required in the proposed approach. Therefore, the inherent distortion of the projection lens can be measured independently without most of systematic errors. In additional, precisely adjusting the projector and optical components before calibration is not a requirement in this approach, which contributes to an easy operation. Finally, the polynomial fitting method is used to represent the projection lens’ distortion character so that the residues are dramatically reduced [14]. All of these improvements lead to high accuracy and high reliability in calibrating the digital projector.

The rest of the paper is organized as follows. In Section 2, the geometry model and the measurement principle are demonstrated in four aspects. In Section 3, experiment system configuration and measurement procedure are detailed. The calibration procedure of the projection lens is also showed in this section and a quantitative evaluation experiment illustrates the final calibration accuracy by using different methods. Finally, Section 4 concludes the paper.

## 2. Method of Distortion Measurement and Correction

### 2.1. Principle and Process of Distortion Measurement

In this paper, the projecting error (distortion) of the projector is considered independently without camera. Thus, the projector’s object and image planes are defined in the way of the projector’s optical design: The plane of the optical modulator (e.g., LCD, DMD) is defined as the projector’s object plane. The definition is opposite to the camera’s image and object planes.

Moreover, the projector’s pixel is used to show the projecting accuracy in the following sections, which does not depend on the dimension of the projecting image. Compared with the metric accuracy, the pixel accuracy more directly describes the projecting lens’ performance of geometrical optics.

**Figure 1:**
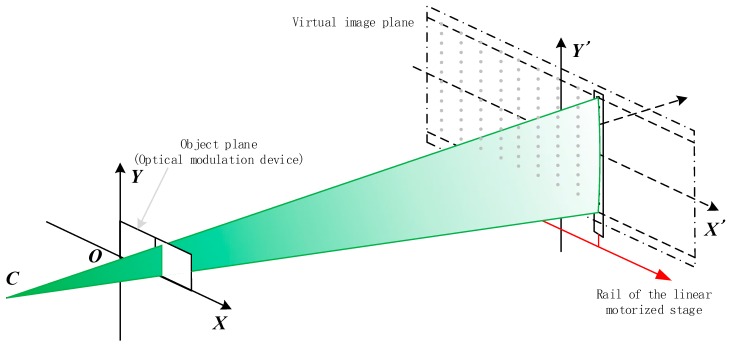
Geometry model of calibration system. The solid square on plane XOY indicates the position of the optical modulator. Point C indicates the optical center and the line COO’ indicates the primary optical axis of the projector in pinhole model. The dashed square on plane O’X’Y’ indicates the motive track of the photoelectric target. The gray points on plane O’X’Y’ are VMPs.

The object plane of a digital projector can be treated as an ideal plane, which is located at the surface of the optical modulation device. A Cartesian coordinate system is built up on the object plane, and each pixel of the plane has a unit size on the coordinate system. Besides, the pattern to be projected is supposed to be ideal on the object plane. However, due to the distortion of the projection lens, the projected pattern on the image plane is deformed. For example, when a straight line on the object plane is projected onto the image plane, it turns out to be a curve. To detect the curve pattern precisely, several PDs contributed to the attainment of this objective. These PDs are precisely fixed along a straight line and named as photoelectric targets. A series of line patterns are projected onto the target and then the corresponding coordinates on the object plane of all the PDs can be calculated from the PDs’ detected results in terms of sequence. The width of each line pattern is one pixel so the measurement process is called pixel scanning measurement, which will be elaborated in the Section 2.2. In order to process through the entire projection image plane, the photoelectric target is driven by a linear motorized stage to stop several times. In each stop of the stage, several Virtual Marker Points (VMPs) are generated as illustrated in Figure 1. All these VMPs make up an ideal coplanar grid. Through repeating the measurement process as mentioned above, the corresponding coordinates on the object plane of all the VMPs can be calculated.

All the VMPs’ coordinates on the image plane are known after precise adjustment, which is detailed in Section 3.1. Compared with the distortion on the image plane, the positioning error of the VMPs is small enough to be neglected. After pixel scanning measurement, the positions of all VMPs are mapped onto the object plane. The mapped positions on the object plane are no longer collinear in X and Y directions due to the distortion of the projection lens. Curves to represent the distortion property are generated by polynomial fitting method for each row and column of the VMPs’ corresponding locations on the object plane. If these curves on the object image are projected onto the image plane, they will be an ideal grid. In addition, higher accuracy can be achieved by increasing the sampling density, *i.e.*, the linear stage stops at more positions. Finally, linear distortion, which is caused by the projector’s posture deviation, has already been considered in the final calibration result so it is not required to adjust the projector’s posture exactly before calibration.

### 2.2. Subpixel Object Plane Coordinates Extraction of the Corresponding VMPs

A pixel scanning method is proposed to extract the subpixel coordinates on the object plane, which corresponds to the VMP coordinates on the image plane.

**Figure 2:**
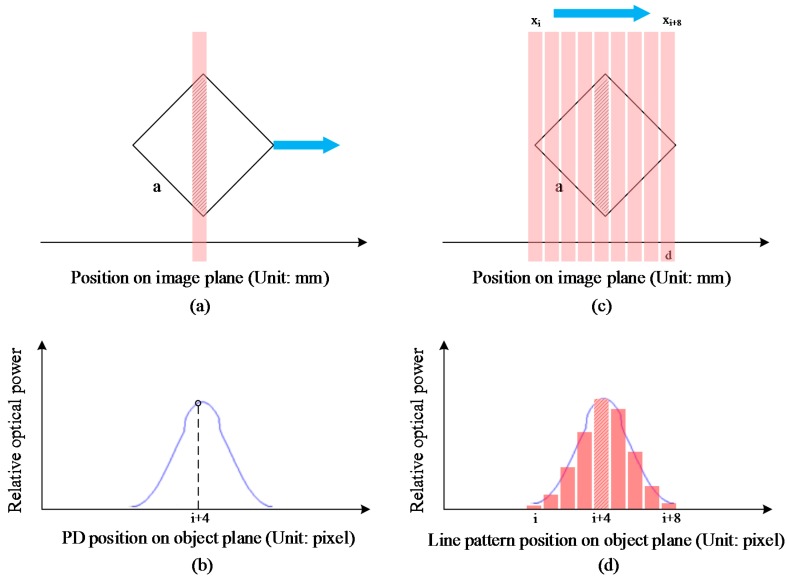
Subpixel coordinates extraction procedure of a VMP. (**a**,**b**) PD output variation law (the blue optic power curve) of a fixed line pattern and a sweeping PD in horizontal direction; (**c**) Scanning process for pixel coordinates extraction. Only the horizontal pixel gap is illustrated. The shaded area shows the highest optical power of this scanning sequence; (**d**) Optical power data of a scanning sequence. The bars show the relative optical power of the scanning sequence. The envelope of the bars is the theoretical optic power curve.

The output of the PD indicates the detected optical power. When an intensity pattern that has uniform distribution is projected on to the PD by a well-focused projector, the PD output is proportional to the size of the illuminated photosensitive area [15]. As shown in Figure 2a, when the PD’s center area is illuminated by the pixel line pattern, the PD output will reach the local maximum value. Thus, the following conditions are presumed to find the corresponding relation between the size of the illuminated and the position on image plane of the PD: First, the width of the line pattern on the object plane is one pixel length; Second, the width of the line pattern on the image plane is narrow enough as shown in Figure 2c, where, $d<\frac{\sqrt{2}}{2}a$ should be satisfied d is the width of the line pattern on the image plane and a is the side length of the photosensitive area). The PD sweeps over the line pattern’s image along the horizontal direction and then the optic power curve is generated. Thus, the corresponding relation between coordinates on the image plane and the coordinate on the object plane is established.

Some further processes are applied to establish the VMP center coordinates and the corresponding VMP subpixel coordinates on the object plane. PD is fixed at a known position on the image plane and then the line pattern sweeps over the PD. To meet the intensity uniform condition, the pixel coordinates of each line pattern should be integers because of the gap between adjacent pixels. Thus, a scan sequence of line patterns is generated and projected on to the PD one by one and the sequential output is collected. The entire process above is named as a pixel column/row scanning process.

When the pixel column scanning process is carried out, a VMP on the projector’s image plane and the corresponding VMP on the object plane are created. The center of the VMP is defined as its own coordinates on the image plane and recorded as *P'(x',y')* (Unit: mm). Meanwhile, the subpixel coordinates of the VMP on the object plane can be precisely estimated from the obtained optic power sequence. Least square fitting method is applied to the obtained optic power sequence with the pre-generated optic power envelope which is shown in Figure 2b. As illustrated in Figure 2d, the central coordinate of the envelope shown as the blue line is the sub-pixel coordinates on the object plane in horizontal direction. The sub-pixel coordinates in vertical direction can be also obtained by pixel row scanning process. The corresponding VMP coordinate on the object plane is recorded as *P(x,y)* (Unit: pixel). With the above approach, the VMP on the image plane is numerically related with its corresponding point on the object plane.

### 2.3. Polynomial Representation of Distortion

**Figure 3:**
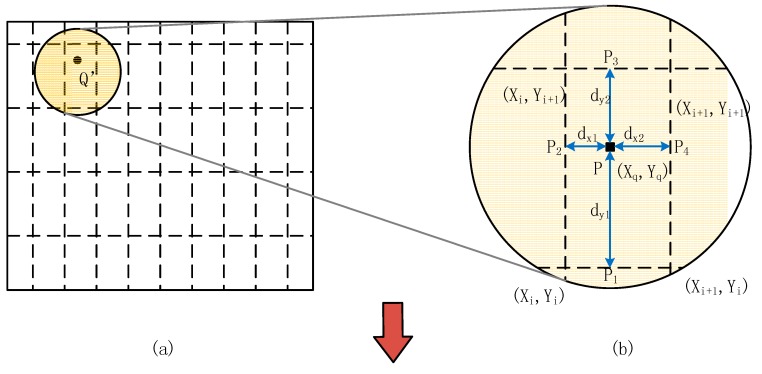

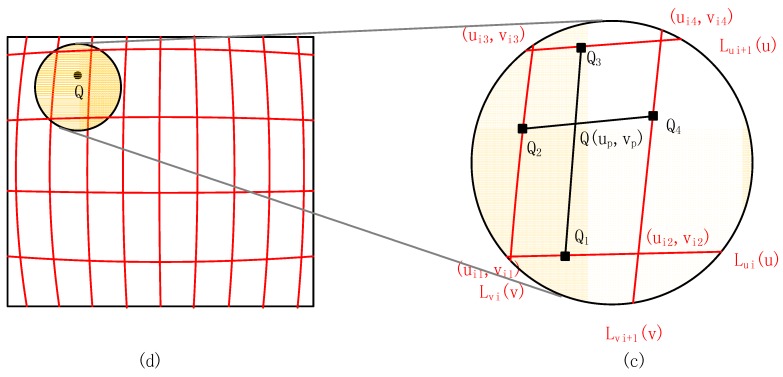
Precise interpolation method in point mapping correction method. (**a**,**b**) The dashed lines indicate the ideal grid created by VMPs in the image plane where, point Q’ is an ideal point of undistorted pattern on the image plane; (**c**,**d**) Red curves show the curves of the polynomial equations. The point Q is the corresponding point on the object plane mapped to the point Q’.

After all the VMPs’ coordinates on the object plane is acquired, some methods should be deployed to represent the distortion property of the projection lens. Approximation with conventional distortion model in entire field of projector makes use of a few parameters to represent the distortion in entire field of view. Instead, in the proposed method based on polynomial representation, one polynomial only represents a small area of the entire field of projector, which contributes to higher accuracy in the distortion representation in terms of smaller residual. In addition, *m* rows and *n* columns of VMPs make up the m×n polynomial equations. As shown in Figure 3, the obtained m×n polynomial equations are displayed as a curve grid on the object plane.

### 2.4. Distortion Correction in Image Space

The objective of projector distortion calibration is to create undistorted patterns on the image plane, and this final step will be detailed in this subsection. Our method is based on the principle that any pattern on both planes can be considered as a finite number of points. Therefore, in order to recover the pattern on the image plane from the object plane, each point at the object plane should correspond to point at the image plane. The procedure is called the point mapping method. The ideal points on the image plane will make up the final ideal pattern after applying this procedure several times.

As mentioned above, a PD at each stop can be treated as a VMP with known position on the image plane and all these VMPs make up an ideal grid. As shown in Figure 1, an orthogonal coordinate system *X'O'Y'* is on the image plane and the stage moves in the direction from *O'* to *X'* along *O'X'*. Any point *Q'* on the image plane has its corresponding point *Q* on the object plane, as shown in Figure 3. An interpolation method is used to precisely calculate the coordinate of point *Q*. First, four adjacent VMPs on the image plane and their four corresponding points on the object plane are obtained. Second, the coordinates of *Q_1_Q_2_Q_3_Q_4_* are obtained. The parameters are illustrated in Figure 3b,c.

Finally, the point *Q* is obtained as the intersection point of line *Q_1_Q_3_* and line *Q_2_Q_4_*. With all the approaches mentioned above, the coordinates of point *Q'* is finally obtained with the knowledge of point *Q* on the object plane.

In conclusion, the sequence of arithmetic to calculate the corrected point $Q\left(x_{Q},y_{Q}\right)$ from the ideal point $P\left(x_{p},y_{p}\right)$ in projector’s image plane is shown as follows (all the names of points and curves can be found in Figure 3).

Find four adjacent VMP coordinates by using the point $P\left(x_{p},y_{p}\right)$ in both image and object plane:

$$\left(X_{i},Y_{i}\right)\;\left(X_{i+1},Y_{i}\right)\;\left(X_{i},Y_{i+1}\right)\;\left(X_{i+1},Y_{i+1}\right)$$

$$\left(u_{i1},v_{i1}\right)\;\left(u_{i2},v_{i2}\right)\;\left(u_{i3},v_{i3}\right)\;\left(u_{i4},v_{i4}\right)$$

Calculate the four corresponding points $Q_{1}\left(x_{Q_{1}},y_{Q_{1}}\right)Q_{2}\left(x_{Q_{2}},y_{Q_{2}}\right)Q_{3}\left(x_{Q_{3}},y_{Q_{3}}\right)Q_{4}\left(x_{Q_{4}},y_{Q_{4}}\right)$ by using four polynomial equations of the adjacent polynomial curves $L_{vi}\left(v\right),L_{ui}\left(u\right),L_{vi+1}\left(v\right),L_{ui+1}\left(u\right)$:

(1) $$Q_{1}\left(u_{i1}+\frac{x_{p}-X_{i}}{X_{i+1}-X_{i}}\left(u_{i2}-u_{i1}\right),L_{ui}\left(u_{i1}+\frac{x_{p}-X_{i}}{X_{i+1}-X_{i}}\left(u_{i2}-u_{i1}\right)\right)\right)$$

(2) $$Q_{2}\left(v_{i1}+\frac{y_{p}-Y_{i}}{Y_{i+1}-Y_{i}}\left(v_{i3}-v_{i1}\right),L_{vi}\left(v_{i1}+\frac{y_{p}-Y_{i}}{Y_{i+1}-Y_{i}}\left(v_{i3}-v_{i1}\right)\right)\right)$$

(3) $$Q_{3}\left(u_{i3}+\frac{x_{p}-X_{i}}{X_{i+1}-X_{i}}\left(u_{i4}-u_{i3}\right),L_{ui+1}\left(u_{i3}+\frac{x_{p}-X_{i}}{X_{i+1}-X_{i}}\left(u_{i4}-u_{i3}\right)\right)\right)$$

(4) $$Q_{4}\left(v_{i2}+\frac{y_{p}-Y_{i}}{Y_{i+1}-Y_{i}}\left(v_{i4}-v_{i2}\right),L_{vi+1}\left(v_{i2}+\frac{y_{p}-Y_{i}}{Y_{i+1}-Y_{i}}\left(v_{i4}-v_{i2}\right)\right)\right)$$

Calculate the length ratio using cross-product:

(5) $$\frac{\left|Q_{4}P\right|}{\left|Q_{2}P\right|}=\frac{S_{△Q_{1}Q_{2}Q_{3}}}{S_{△Q_{1}Q_{4}Q_{3}}}=\frac{\left|\vec{Q_{1}Q_{2}}\times \vec{Q_{1}Q_{3}}\right|}{\left|\vec{Q_{1}Q_{4}}\times \vec{Q_{1}Q_{2}}\right|}$$

Calculate the $P\left(x_{p},y_{p}\right)$ using the formula for definite proportional division point:

(6) $$x_{q}=\frac{S_{△Q_{1}Q_{2}Q_{3}}\cdot x_{Q_{4}}+S_{△Q_{1}Q_{4}Q_{3}}\cdot x_{Q_{2}}}{S_{△Q_{1}Q_{2}Q_{3}}+S_{△Q_{1}Q_{4}Q_{3}}}$$

(7) $$y_{q}=\frac{S_{△Q_{1}Q_{2}Q_{3}}\cdot y_{Q_{4}}+S_{△Q_{1}Q_{4}Q_{3}}\cdot y_{Q_{2}}}{S_{△Q_{1}Q_{2}Q_{3}}+S_{△Q_{1}Q_{4}Q_{3}}}$$

## 3. Experiments and Results

### 3.1. Experiment Setup

**Figure 4:**
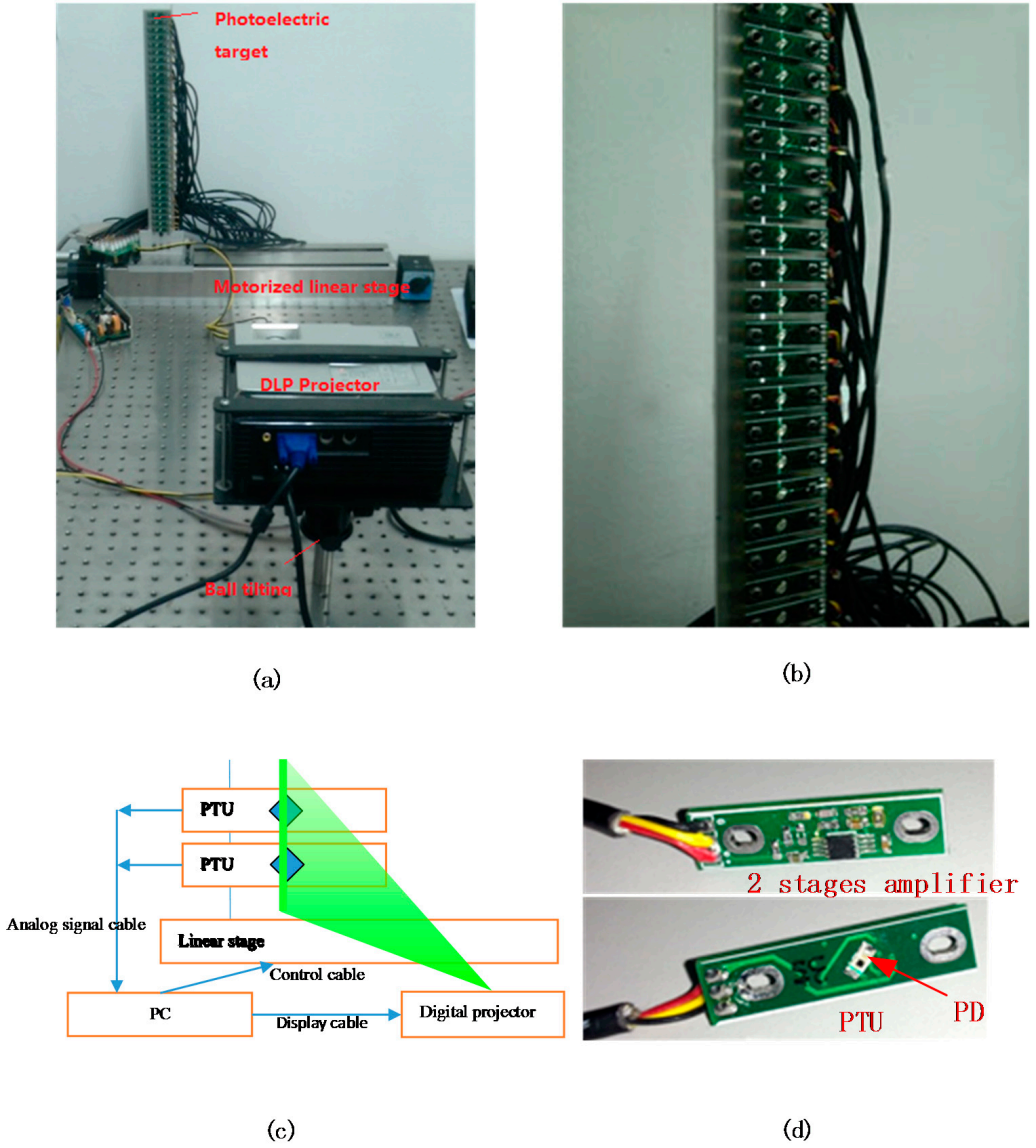
Experiment setup of the calibration system. (**a**) Relative positions of the DLP projector, the photoelectric target and the motorized stage; (**b**) The photoelectric target with the projected line pattern; (**c**) Schematics of the entire projector calibrating system (Only two PTUs are shown) and (**d**) the enlarged view of the photoelectric module.

The experiment setup consists of a photoelectric target, a motorized linear stage, a projector (Model: BENQ CP270) and its posture adjustment mechanism (a ball tilting) as shown in Figure 4a. The electronic control subsystem (a computer) and some extension cards were applied to drive the motorized stage, collect the voltage signals and control the projector (not shown in Figures). PDs (Model: Osram SFH2701) were mounted on the front of the photoelectric transformation units (PTUs), as shown in Figure 4b. At the back of the PTU, a two-stage amplification circuit (transimpedance amplifier circuit) was applied to convert the photocurrent signal of the PD to voltage signal and then a multichannel data collection card (Model: Advantech PCI-1713U 32 channels) was used to store all the amplified signals from the 32 PDs. The number of the PTUs depends on the installing density and the height of the projected image. To capture the pixel lines in horizontal direction, the total height of the photoelectric target should be longer than the height of the projected image.

The installation reference line of the PTUs should be perpendicular to the movement direction of the stage in order to ensure the orthogonality of the coordinate system on the virtual image plane. The perpendicularity between the stage and the photoelectric target was guaranteed by precise machining and redundant locating. All the coordinates of PTU locating holes and fixing holes can be obtained by a measuring microscope (Model: TESA VISIO 300 DCC) and then the reference line for PTU installing was generated. An “L”-shaped mechanical component was designed to ensure the perpendicularity between the slide stage and the photoelectric target and then the installation perpendicularity of photoelectric target can be guaranteed within ±20 arc seconds.

**Figure 5:**
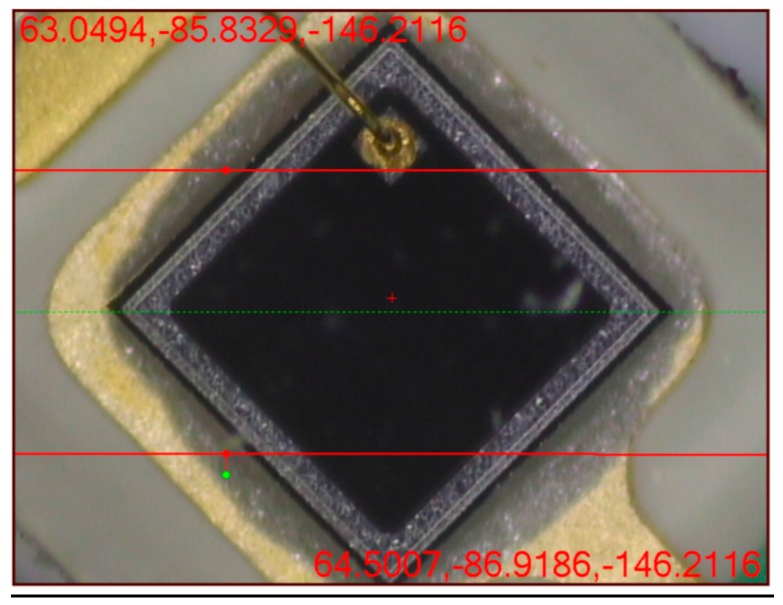
Photodiode assembly under image measurement tool. The green dotted line is the reference line for photodiode assembly. Numbers in the figure indicate the coordinate of the image corner point. (XYZ, Unit: mm).

The measuring microscope was also applied to make all the 32 PTUs collinear and equidistant through fine adjustment. The PD was installed on the surface of the PTU with 45° with the horizontal direction. The photosensitive area of the PD is a square and the side length is 0.6 mm so the length of PD’s receiving area is 0.84 mm (diagonal). With the help of the adjustment screws on the photoelectric target, the installation straightness of all the 32PTUs can be guaranteed within 0.008 mm. Figure 5 shows the image of the photodiode under the measuring microscope.

The stage which holds ±10 *μm* positioning accuracy and ±3 *μm* repetitiveness was calibrated by a laser interferometer (Model: Renishaw XL-80) to ensure the accurate positioning along the travelling line. We only employ unidirectional motion during one calibration procedure to avoid the backlash of the linear stage, so the final positioning accuracy of the stage can be guaranteed within 0.006 mm in a travel distance of 300 mm.

The projector to be calibrated has a resolution of 1024 × 768. In order to test the proposed approach independently, all the projector’s pre-included geometric correction functions were turned off. The projector was fixed on a ball tilting and the projector’s control panel was facing upwards to make sure the origin point of the object plane coordinate was located at the bottom right. The optical axis does not need to be exactly perpendicular to the virtual image plane because the linear distortion parameters have already been considered in the final calibration results in the proposed method.

A motion controlling card (Model: Advantech PCI-1240) programmed by the computer was used to control the motorized stage. A data collection card (Model: Advantech PCI-1713U) programmed by computer recorded the voltage values representing the optical power. The line patterns for the projector were generated by the computer. The graphics card of the computer was set up to drive two display devices simultaneously, one for a LCD monitor and the other for the DLP projector.

A horizontal coordinate list for the stops is generated by the computer with fixed distance in between. The number of stops can be increased to achieve higher accuracy. In each stop, the computer records the current position of the linear motorized stages, the scanned sequence as mentioned in Section 2 and output voltage values of the PD. The measured data were stored in the computer for post-processing by MATLAB.

Another model of a projector with different resolution (Model: CASIO XJ-M255 1280 × 800) is applied to test and verify the proposed calibration method, which is only shown in the supplementary attachment (Figures S1–S5).

### 3.2. Measurement Results

The vertical distance of two adjacent PTUs is 10 mm while the horizontal distance between two stops of the motorized stage is 8 mm. The width of projecting image was adjusted to be 280 mm by changing the working distance. The projector’s width-height ratio is 4:3 so the height of the projected image is about 220 mm (a little longer than 210 mm) and only 21 PTUs are used in this calibration procedure. The image width of one pixel is approximately 0.27 mm to meet the accuracy requirement. The calibration procedure costs about 40 min when the speed of the procedure is optimized.

**Figure 6:**
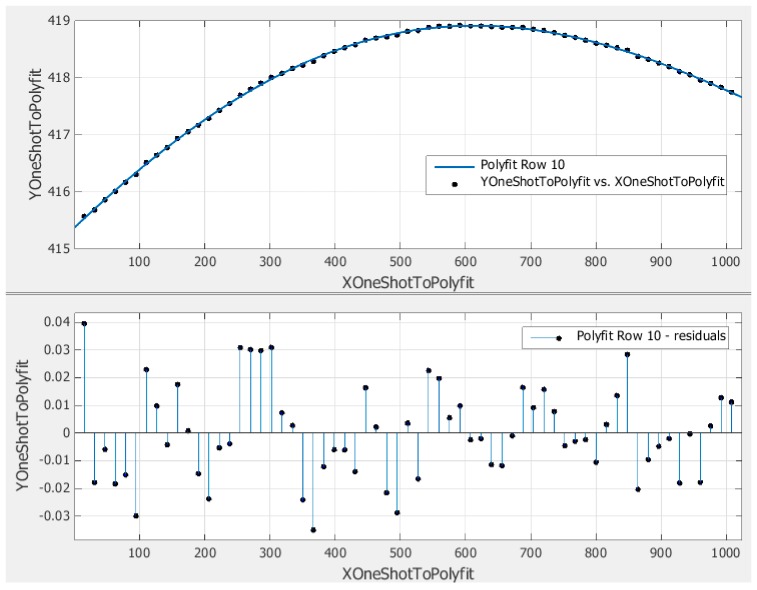
Final evaluation results of the polynomial fitting.

Figure 6 shows the polynomial fitting evaluating result. Before fitting all the rows and columns of the VMPs, the degree of the polynomial should be determined. To simplify the data processing, 2–6 degree polynomials are tried to fit Row 10 of the VMPs in order to evaluate the accuracy of the fitting. Finally, the polynomial with degree 4 is chosen because it has the lowest RMSE while the coefficients are not less than 1 × 10^−14^ (limit of the double precision float number).

**Figure 7:**
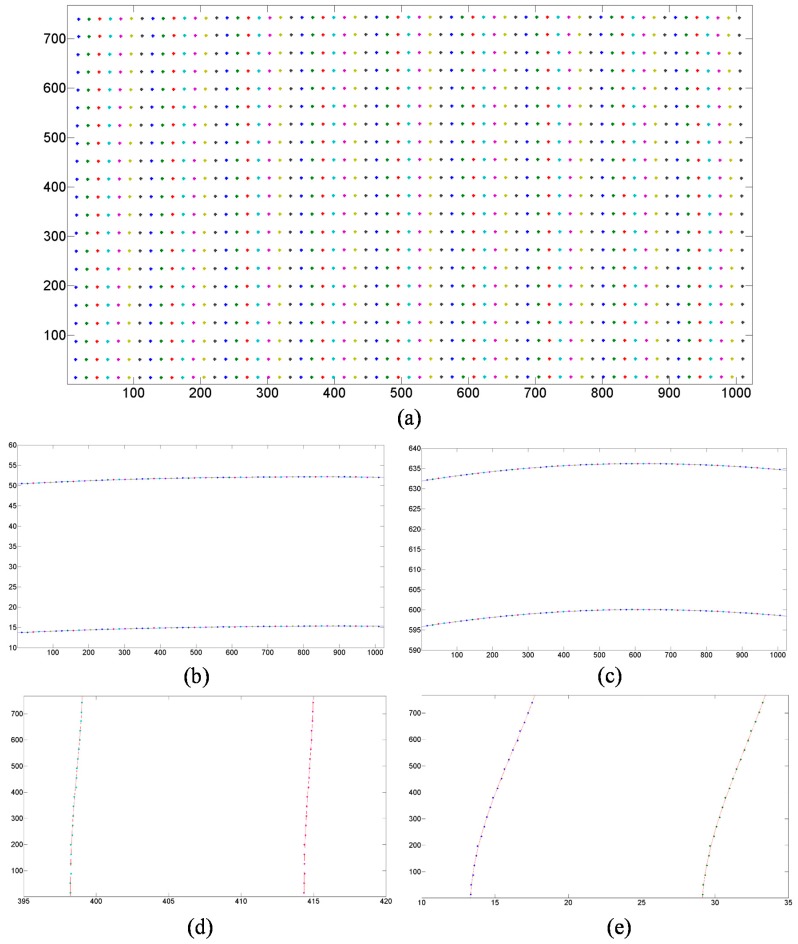
Results after obtaining VMPs’ coordinates and polynomial fitting (unit: pixel). (**a**) The points on the object plane corresponding to the VMPs on the image plane; (**b**) and (**c**) give the partial enlarged views of the Axis Y; (**d**,**e**) give the partial enlarged views of the Axis X. The curves indicate polynomial curve of these measuring points.

Figure 7 shows the results after obtaining mark point coordinates and applying polynomial fitting on the object plane. The results in Figure 7 show the distortion property of the projecting lens intuitively. Because the tested projection lens has an off-axis optical path, the distortion is up-down asymmetry. All the VMPs and polynomial curves in the figures show an obvious barrel distortion when the tested lens is used as the camera lens. In fact, a pillow distortion will appear when an ideal pattern is projected onto the image plane with this tested projection lens. The maximum non-linear distortion of the projector which appears at the top of the projection image is about three pixels.

### 3.3. Quantitative Evaluation

The calibration purpose of projection lens is to eliminate the non-linear distortion. If a corrected line pattern is projected onto a flat plate, the image of this line pattern should be straight. The performance of the calibrated system can be quantitatively evaluated by comparing with that of an uncorrected line pattern.

**Figure 8:**
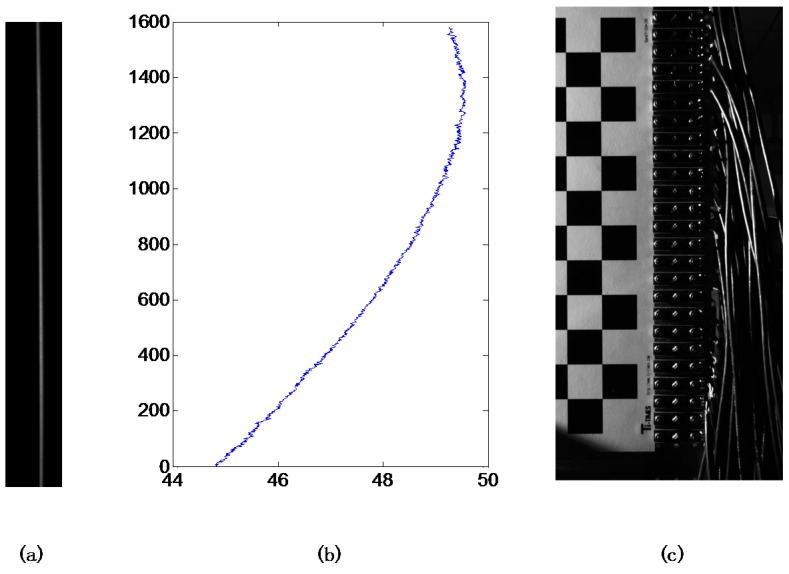
The line images captured by the CCD camera. (**a**) Image captured by the camera; and (**b**) Line pattern center extraction result of all the image rows (Unit: pixel); (**c**) Image of the checkerboard calibration plate.

A low noise, precise imaging system is built up to evaluate the projector calibration system, which makes use of a high resolution CCD camera (Imperx B2520M, 2456 × 2058 pixels, BW) and a white plate with flatness error smaller than ±5 micrometers. The accuracy of this evaluation is affected by distortion of the camera lens. To minimize this effect of camera lens distortion, the CCD’s central area was used to record the image of the line pattern, as illustrated in Figure 8b. The line pattern mapped from the ideal line on the image plane has a sequence of non-integral coordinates on the object plane. To maximize the evaluation accuracy, Gaussian function is used to generate the corrected line pattern to project with subpixel accuracy. Then, the subpixel coordinates of the captured line is also extracted by Gaussian function fitting method to determine the center position sequence of the projected line pattern.

The distortion is more severe at the boundary area of the projected image, so pixel columns near the edge of the projectored image were evaluated to validate the proposed method. Three kinds of line patterns were generated and projected onto the plate surface for comparison.

1. Uncorrected straight line: Pixel column 17 (Pixel columns are 1–768 from left to right).
2. Corrected line by model-based method as shown in Reference [8]: Like the distortion correction of a photograph, the distortion correction of a projector can be achieved by rearranging the pixels on the projector’s object plane with the method based on conventional distortion representation. The ideal line (goal of the correction) on the image plane is the pixel column 17 in the linear (pinhole) model.
3. Corrected line by the proposed polynomial distortion representation: The ideal vertical line (goal of the correction) on the image plane is located at 17/768 of the image width.

**Figure 9:**
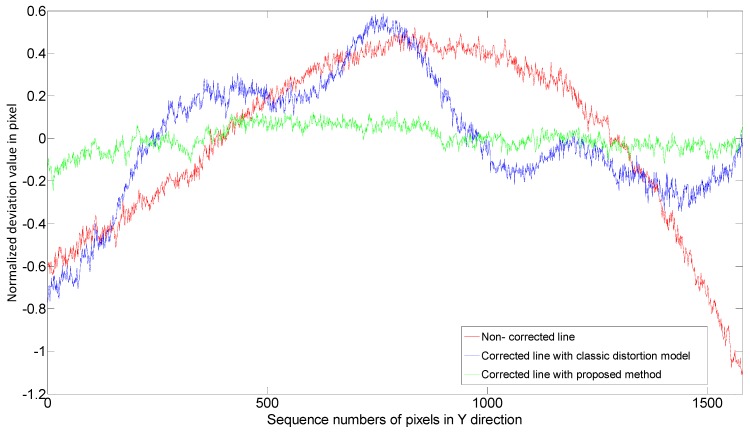
Evaluation results on non-linear residual.

All these three line pattern images were captured by the CCD camera. The center coordinate sequences of the line patterns were extracted by Gaussian fitting method from the captured images. Each sequence should be corrected by the reference line sequence that was generated previously. In order to show the correction performance on the non-linear deviation, the linear components of the three sequences should be removed. To calculate the deviations in metric, a ceramic checkerboard calibration plate is used to generate a metric reference. The distance between each corner point is 20 mm on the plate and 150 camera pixels in the photograph (Figure 8c) so a camera pixel stands for 0.133 mm. The final results of projection distortion correction were illustrated in Figure 9. In fact, the average non-linear deviations of the three images were 0.35, 0.24, and 0.05 pixels (0.047, 0.032, 0.007 mm) respectively, while the maximum non-linear deviation were 1.63, 1.36 and 0.38 pixels (0.217, 0.181, 0.051 mm) respectively. All the metric deviations are calculated with a projecting image dimension 280 × 210 mm. The experimental results clearly showed that the proposed method accurately calibrates the projector distortion characters and the corresponding corrected line effectively decreases the distortion.

## 4. Conclusions

This paper proposes a projection lens distortion calibration method based on PD detection and polynomial representation. A pre-calibrated scanning system has been built up to precisely detect the ray emitted from a projector. A pixel scanning procedure and curve fitting approach are used to directly obtain the ray’s coordinates on the projector’s object plane. Applying the polynomial fitting method to the obtained coordinates helps to approximate the distortion of the projecting optical system’s accuracy. Compared with the method based on camera imaging and classic distortion models, the proposed method has smaller residual. Experimental results show that the proposed method is able to provide a more precise distortion representation than the existing methods with camera. The proposed method is suitable for some accuracy sensitive applications (*i.e.*, some measuring sensors that can be treated as an integrated product). In addition, the proposed way to track the position of the rays in free space can be used in other applications to evaluate the quality of the optical system independently and precisely.

## Supplementary Files

Supplementary File 1
