# Supplementary material for: An Accurate Projector Calibration Method Based on Polynomial Distortion Representation
Source: Sensors (Basel). 2015 Oct 20;15(10):26567–82. doi: 10.3390/s151026567 (PMC4634452; doi:10.3390/s151026567)
Supplement: Supplementary File 1 [file sensors-15-26567-s001.pdf]

OPEN ACCESS
sensors
ISSN 1424-8220
www.mdpi.com/journal/sensors

Supplementary Information

An Accurate Projector Calibration Method Based on Polynomial Distortion Representation. *Sensors* 2015, *15*, 26567-26582

Miao Liu [1], Changku Sun [1], Shujun Huang [2] and Zonghua Zhang [2,*]

[1] State Key Laboratory of Precision Measuring Technology and Instruments, Tianjin University, Tianjin 300072, China; E-Mails: excalibur_s@tju.edu.cn (M.L.); sunck@tju.edu.cn (C.S.)

[2] School of Mechanical Engineering, Hebei University of Technology, Tianjin 300130, China; E-Mail: huangsj@hebut.edu.cn

* Author to whom correspondence should be addressed; E-Mail: zhzhang@hebut.edu.cn; Tel./Fax: +86-22-2658-2403.

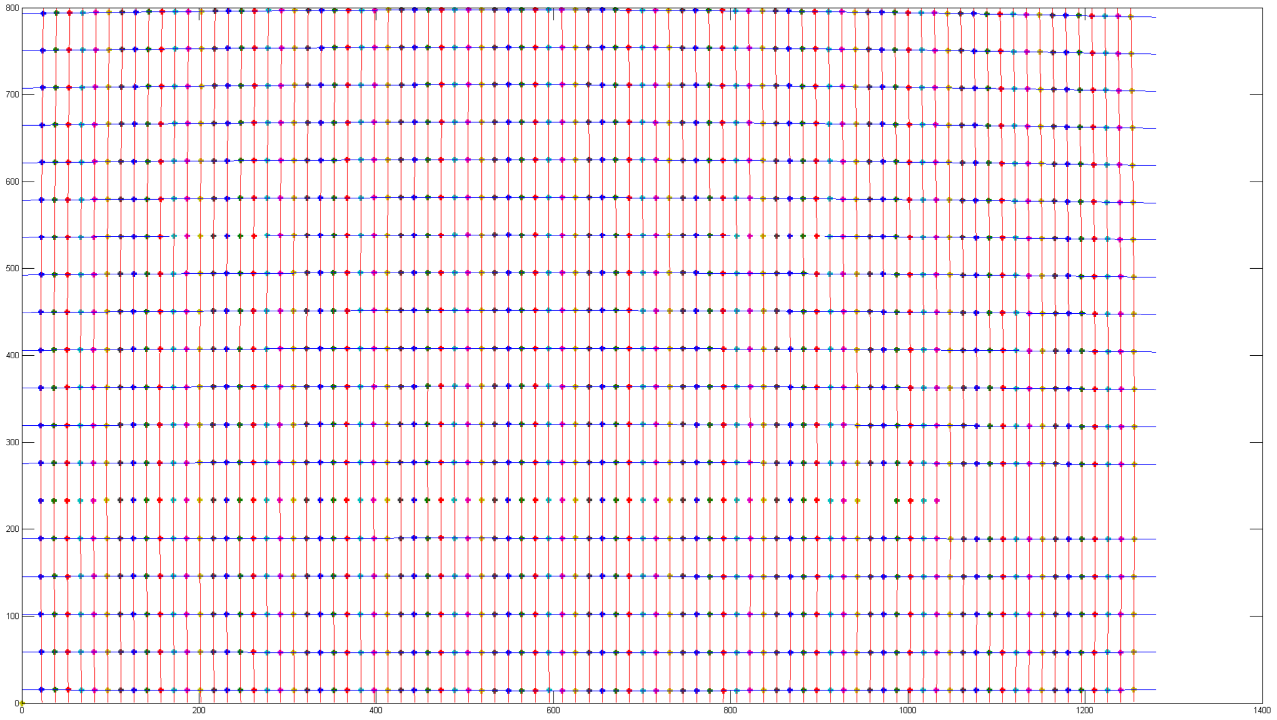

Figure S1. Distortion_Map_XJ-M255_overall.

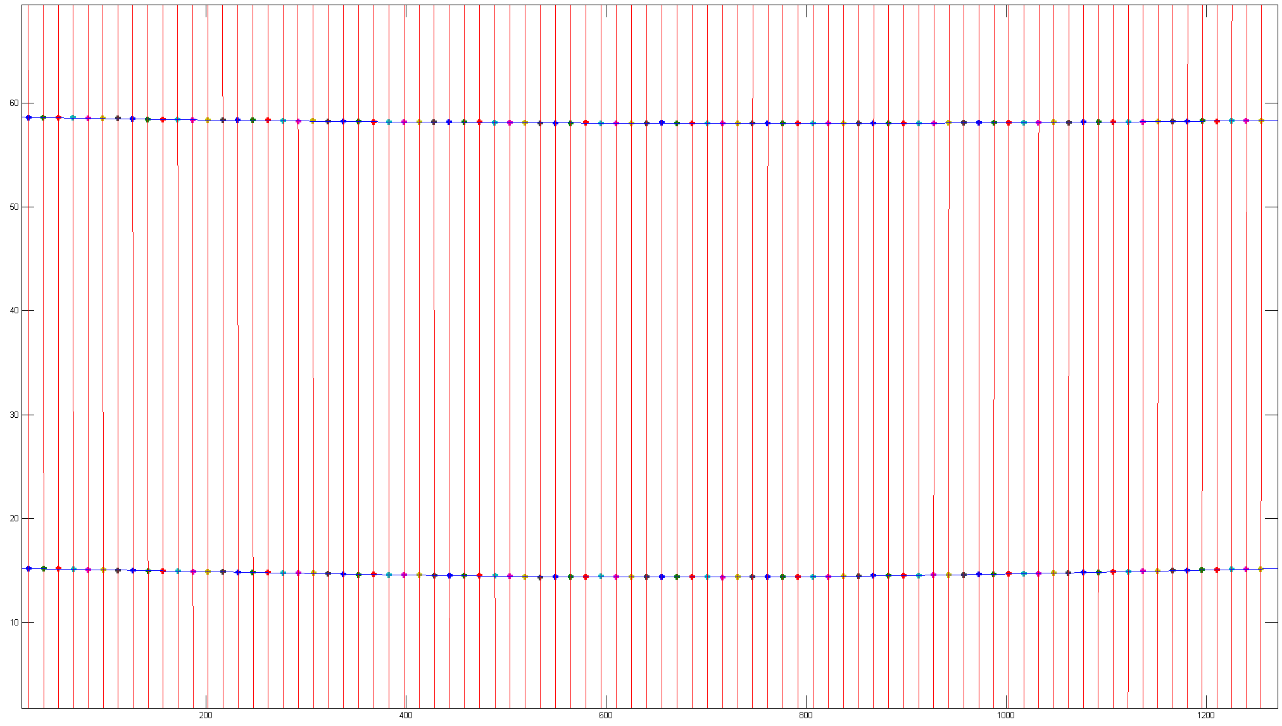

Figure S2. Distortion_Map_XJ-M255_bottom.

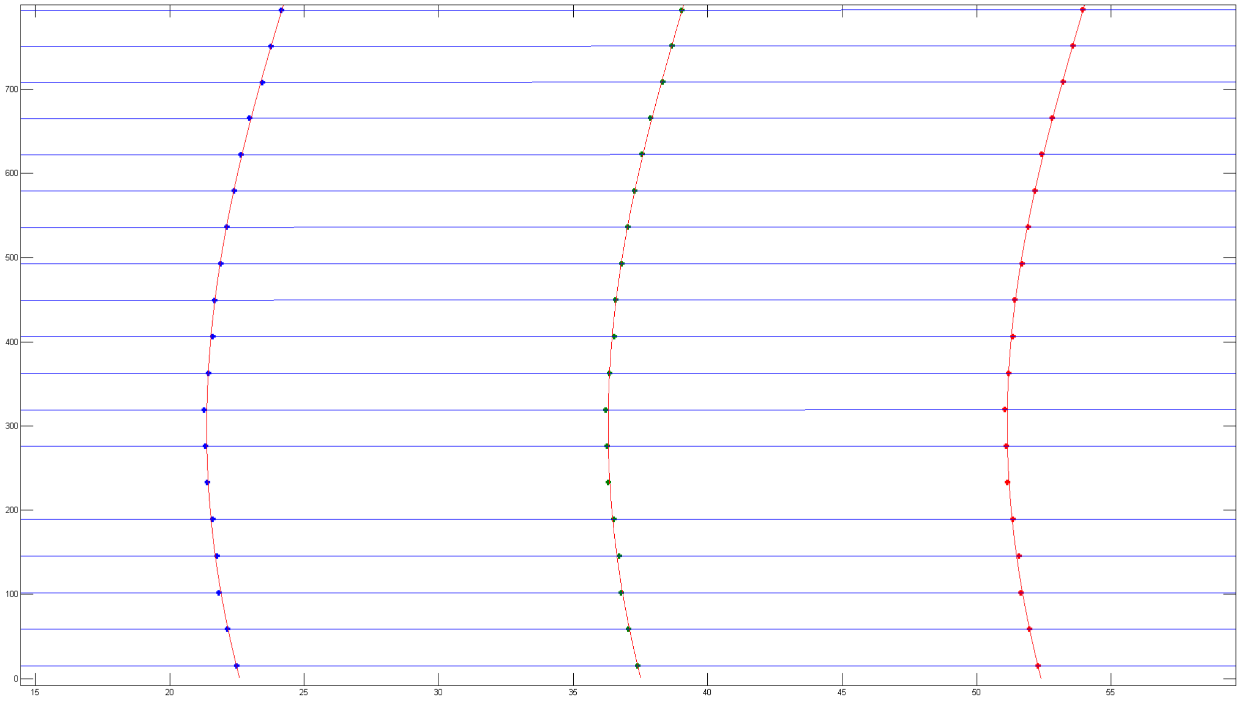

Figure S3. Distortion_Map_XJ-M255_left.

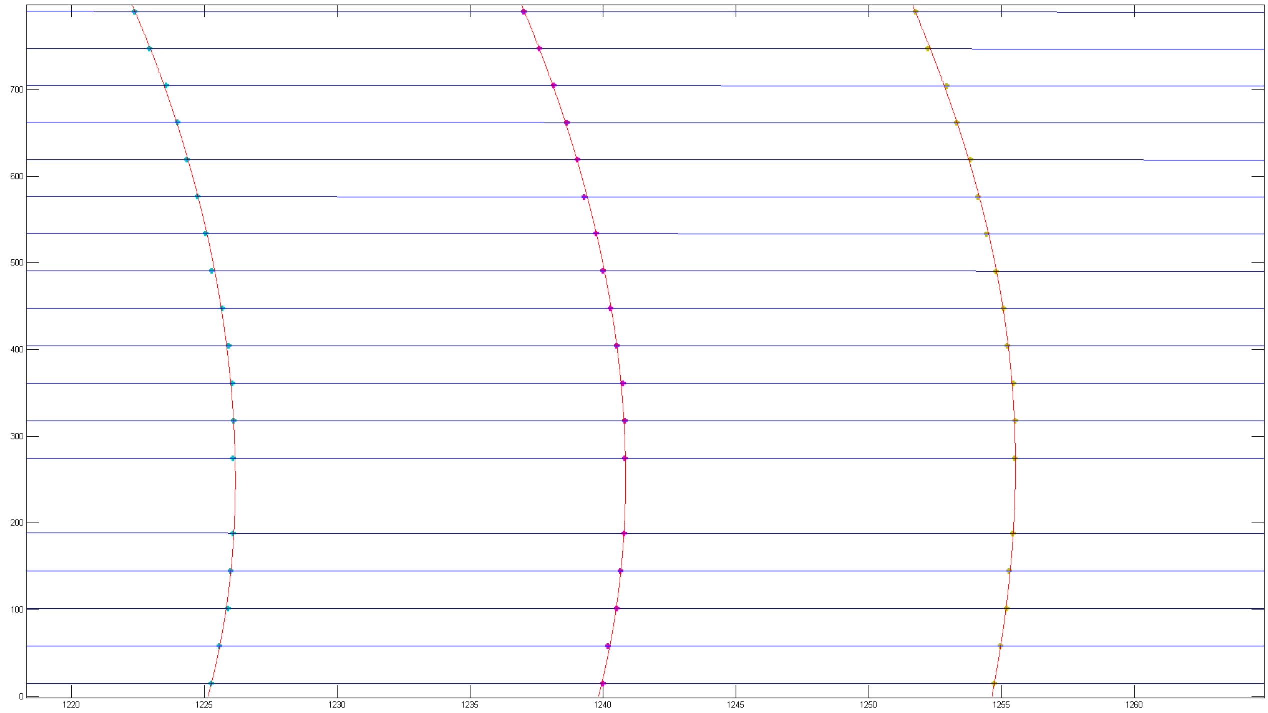

Figure S4. Distortion_Map_XJ-M255_right.

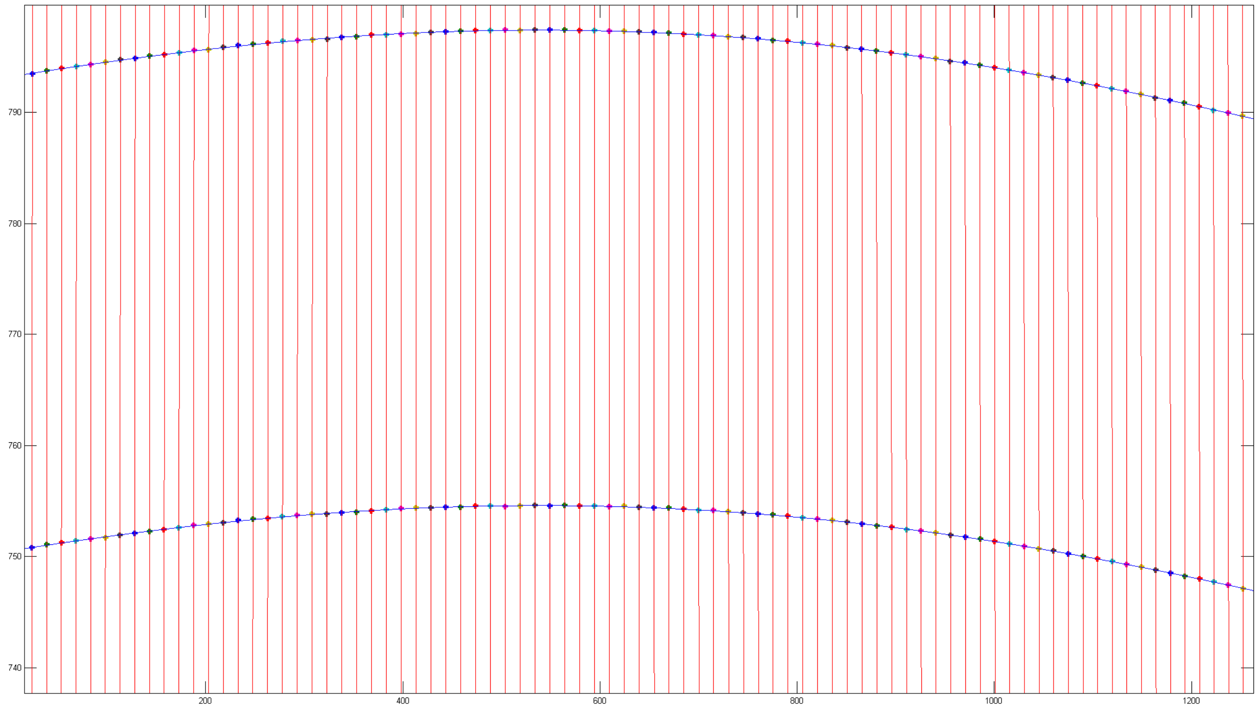

Figure S5. Distortion_Map_XJ-M255_top.

© 2015 by the authors; licensee MDPI, Basel, Switzerland. This article is an open access article distributed under the terms and conditions of the Creative Commons Attribution license (http://creativecommons.org/licenses/by/4.0/).
